# Examining the paradoxical effects of kratom: a narrative inquiry

**DOI:** 10.3389/fphar.2023.1174139

**Published:** 2023-05-05

**Authors:** Kirsten E. Smith, Jeffrey D. Feldman, Kelly E. Dunn, Christopher R. McCurdy, Stephanie T. Weiss, Oliver Grundmann, Albert Garcia-Romeu, Janeen Nichels, David H. Epstein

**Affiliations:** ^1^ Real-World Assessment, Prediction, and Treatment Unit, National Institute on Drug Abuse Intramural Research Program, Baltimore, MD, United States; ^2^ Department of Psychiatry and Behavioral Sciences, Johns Hopkins University School of Medicine, Baltimore, MD, United States; ^3^ Department of Medicinal Chemistry, College of Pharmacy, University of Florida, Gainesville, FL, United States

**Keywords:** kratom, emerging drugs, novel substances, kratom tolerance, opioids

## Abstract

**Introduction:** Surveys and case reports have documented kratom use in the United States (US) for over a decade. However, those reports have generally not examined in depth the role kratom plays in the lives of those who use it regularly for sustained periods. Until there are controlled studies of the pharmacology and subjective effects of kratom alkaloids in humans, one of the best sources of insight on kratom-product use remains qualitative data with nuanced descriptions of kratom effects from those who use it regularly.

**Method:** We conducted semistructured qualitative interviews with adults who regularly use kratom products, as part of a laboratory study of kratom-product self-administration. This qualitative component of the study was conducted as a narrative case-report series (*n* = 10).

**Results:** Despite some differences among participants, all experienced acute combination effects that were largely, even simultaneously, analgesic and stimulatory. Most participants had decreased their dosages over time, and one planned to quit. Five of the 10 participants met DSM-5-based criteria for kratom-use disorder (3 mild, 1 moderate, 1 severe, by symptoms counts). When kratom was inadvertently taken in larger than intended doses, participants described a constellation of symptoms that they called “the wobbles” (a jittery feeling accompanied by what seemed to be nystagmus); this was rare, but could be of scientific and clinical interest as a possible manifestation of serotonin syndrome. Most participants described tolerance but considered kratom generally safe at low-moderate doses, providing perceived benefits with less potential risk for adverse effects compared to pharmaceuticals or illicit drugs.

**Discussion:** In-depth interview data like these help confirm and clarify findings from larger survey studies and clinician-driven case reports. They are needed to inform the policy practice regarding kratom and may also help inform future experimental designs.

## 1 Introduction

The commodification of the botanical *Mitragyna speciosa* as kratom products in the United States (US), and their increased availability to consumers, has contributed to a rise in use among a diverse population with myriad substance use histories (Covvey et al., 2020; Rogers et al., 2021; Smith et al., 2022a; Grundmann et al., 2022; Smith et al., 2022). Although sharing some characteristics and motivations for use, there are seeming subpopulations of people who use kratom products regularly. These have not been well characterized, partially because most human kratom research comprises survey and case reports of people taking various products marketed as “kratom,” that vary with regard to alkaloid content and possible adulterants or contaminants (Lydecker et al., 2016; Garcia-Romeu et al., 2020; Smith et al., 2022b; Grundmann et al., 2023; Weiss and Brent, 2023). While self-report has provided some insight into use patterns, and case reports have provided details of adverse effects (Smith K. E. et al., 2023; Feldman et al., 2023), the nuanced story of kratom use and motivations in the US is still largely uncharacterized. Indeed, few qualitative data on kratom use exist, and none provide narrative accounts of changes in use over time (Swogger et al., 2015; Smith et al., 2021; Tobacyk et al., 2022). Phenomenological descriptions of kratom’s subjective effects have also not been documented in scientific literature, leaving untapped a basic source of information to help guide the design of controlled experimental studies investigating kratom alkaloids.

### 1.1 Aims

To address this, we conducted in-depth qualitative interviews with kratom-using adults who use regularly as part of a laboratory study investigating kratom product self-administration. Findings from the self-administration study will be published elsewhere. This qualitative component was conducted to characterize participants’ kratom use, from initiation to present day, as a narrative case-report series.

## 2 Materials and methods

Between July and November 2022, ten kratom-using adults who agreed to be recontacted for future research while participating in our nationwide ecological momentary assessment (EMA) study on kratom were enrolled into this smaller pilot substudy that, among other activities, included a qualitative component. To be eligible, participants must have completed the EMA study, live within 150 miles of our Baltimore, MD clinic, and report regular (≥3 times weekly for ≥4 weeks) kratom use. All participants provided voluntary informed consent to participate in this study and have their data summarized in case report form. This study was approved by the National Institutes of Health Institutional Review Board.

A narrative inquiry approach, via semistructured interviews, was chosen for collecting and analyzing qualitative data because our goal was to understand lived experiences of US adults who use kratom. Our interview guide (see Supplemental Material) was developed to allow maximum space for participants to share experiences and stories without presuppositions on our part other than that we were asking participants to describe the evolution of use of a psychoactive substance that is still relatively novel to the US. Our interview guide can be adapted for investigating other emerging substances. Follow-up questions were asked based on participant responses and informed by the content expertise of the interviewer. All interviews were conducted in-person by KES, digitally recorded, and transcribed. Content highlighted here was selected by agreement among the research team. As the objective of this narrative inquiry was not to build theory or generate themes, but rather to treat each story individually, no thematic coding was conducted (Creswell et al., 2007; Creswell and Poth, 2016).

## 3 Results

Table 1 shows participants’ demographics characteristics, and Table 2 presents participants’ kratom dosing patterns. Salient details from interviews are presented below. To the fullest extent possible, we use direct quotes, so participants’ stories can be told in their own words. In addition to the cases and participant quotes presented, we also documented participants’ responses to the issue of whether they would continue to use kratom if it were to become illegal. Those reactions appear in Table 3.

**TABLE 1:** Participant demographic characteristics.

Participant	How did you learn about the study?	Sex	Age	Age kratom use initiation	Race	Education	Employment
1	Reddit	Female	60	55	White	Associate’s Degree	Retired
2	Reddit	Female	26	25	White	Bachelor’s Degree	Part-time
3	Twitter	Male	45	39	White	Master’s Degree	Full-time
4	The American Kratom Association	Male	49	46	White	Bachelor’s Degree	Part-time
5	Facebook; An online forum	Female	35	29	White	Some college	Full-time
6	Facebook	Male	34	25	White	PhD	Full-time
7	Facebook	Male	52	50	White	Bachelor’s Degree	Full-time
8	Friend or family	Male	32	18	Asian, White	Some college	Full-time
9	Friend or family; Instagram	Female	41	36	White	Bachelor’s Degree	Student
10	Facebook	Male	38	27	White	High school/GED	Full-time

**TABLE 2:** Kratom dosing patterns.

Participant	Longest period regular kratom use	Typical dose^a^	Typical doses per day	Lowest-highest effective dose range^b^	Dose that has produced undesired effects^b^	Time to first kratom dose after waking
1	5 years	1 g	5	1–2 g	4 g	6–30 min
2	2 years	3 g or 3 capsules	2	2–8 capsules	10 capsules	6–30 min
3	6 years	1 teaspoon or 3 capsules	2	1–2 teaspoons	3 teaspoons	>60 min
4	8 months	3 g or 3 capsules	3	1.5–3.75 g	4.5 g	6–30 min
5	2 years	4 capsules or 2 mL extract	3	3–5 capsules	6 capsules	31–60 min
6	8 years	2 g	2	3.5–6 g	7 g	6–30 min
7	2 years	10 g	2	7–14 g	20.25 g	31–60 min
8	3 years	8 g or 1.25 tablespoons	2	0.75–1.5 tablespoons	2 tablespoons	>60 min
9	6 years	5 g	3	3–5 g	6.5 g	>60 min
10	7 months	1.5 spoonfuls	1	1–2 spoonfuls	2.75 spoonfuls	Within 5 min

^a^ In an online survey several weeks before the interview, participants were asked to report their kratom dose in the unit or units in which they typically took it (e.g., capsules, spoonfuls). Typical dose reflects more than one unit type in for 5 participants.

^b^ Participants reported these doses or dose ranges in terms of the dosing unit they most often used.

**TABLE 3:** Participants’ responses when asked what they would do if kratom became illegal.

Participant	
**1**	*“I mean, I would go off of it. I don’t think it would change my life dramatically. I think that I would be disappointed if it wasn’t available to other people who really need it more than me. I’ve, you know, I like the way it makes me feel. I like I get more done when I’m using it, especially after that second dose when I’m really feeling energetic and ready to go. But, um, I think there’s a lot of people that can benefit from it…I think it would be a damn shame for, you know, [kratom] to be made illegal across the board without, you know, really doing some substantial studies. Because they’re writing it off without even really giving it, you know, any kind of legitimate case studies…I think it would be beneficial to, you know, um, a big study because I have…it’s like, on one side, I want this to go through like every legal channel possible, but on the other hand, I hate the thought that Big Pharma would get hold of it and then, you know, it’s all about the profit to them, I feel, I truly believe that this--like the lobbyists don’t want kratom, you know, available to people because it get--keeps them in their pockets…keeps the, uh, pharmaceutical companies making big bucks.”*
**2**	*“There’s one more [botanical] called Herosa. There’s a cousin to Kratom called, uh, Mitressa Harostia. Yeah, I’d try something else or, like, Blue Lotus. I would try it, but if I had to, I would find a means to get it. Yes, I would still try. I would still like to figure out how to get it.”*
**3**	*“Now, if something was to--obviously, I mean if, in the Fall, if the DEA would have scheduled it, I would have stopped. So basically, if it’s not broke, don’t fix it. Like, I…I to date have had no negative impact so why stop? I mean, it’s working for me. But if something was to happen, if it was going to be scheduled, then I would stop.”*
**4**	*This participant did not directly answer the question.*
**5**	*“Well, I guess it would be back to the drawing board back where I was in 2016. Where I would try to do more research, maybe try to find a different alternative. Uhm, but I guess, if I had to resort, I would go to a doctor. But I would rather find another route. So, I would probably do some more research and see if there’s anything else out there. Uhm, but like hopefully that never happens, but you know that’s crossed my mind before too. They were under fire back a few years ago. Cause I actually went to the rally in DC. It would [ultimately] just be too much of a hassle. I mean, uhm. You know, you’re just going through a bunch of red flags getting caught. I just don’t think I could be safe. I just don’t think I’d have the patience for something like that. I’m too old. [laughs].*
**6**	*“I don’t think it should be scheduled. I don’t think it should be prohibited. I think we should be able to import it to America and then it needs to go through some kind of screening, for, like contaminants or whatever. Then it needs to be packaged in a way that’s, um, not enticing to, like, people who want to be, like, self-medicating…you shouldn’t be able to buy, like a kratom shot at the gas station…I remember vividly one of the first kratom products I bought was literally called ‘Kratocet’. It sounded like ‘Percocet’.”*
	*Note: As this participant was in the process of quitting, he was not asked if he would still use kratom if it became illegal.*
**7**	*“I’d probably still buy it. Yeah.”*
**8**	*“That’s a really…tricky question to answer. It would depend on a lot of things. It would mostly depend on what its availability and price would be after something like that happens. If it became really hard to get and really expensive, I would stop using it: I don’t need it that much. But because of its availability and legal status and the benefits it offers to me, I want to keep using it, because there aren’t really that many risks at that level.”*
**9**	*“I’d go right about my business like I have with every other illegal drug in my life! (laughs). I would keep getting it, until it… I mean, I dunno, I guess I’m not a millionaire, so cost does matter, and if it got crazy-crazy—but yeah, I don’t generally make decisions based on that kind of thing. So if I was going to quit, it would be for reasons that were not that [laughs]. Well, I have written many letters to Congress, or different states, because I feel like a ban in one state is inching toward a ban in all states, so I’m pretty involved in that, I just really tell my story.”*
**10**	*“I’d probably still take it. I probably would. I would probably just take a lot less, less often [if the price increased]”.*

### 3.1 Participant 1: *“…I do find it helps a bit for pain, but I more so find that it gives me energy.”*

Participant 1 is a 60-year-old female who learned about kratom online when searching for alternatives to opioids for cancer-related pain. Initially, she used kratom for analgesia and to supplement the opioids she had been prescribed for 12 years. Recently she had used kratom to taper off opioids and succeeded in doing so over 6 weeks; she had not used oxycodone products for 2 months. She acknowledged that although she had needed opioids for analgesia, she also enjoyed their effects, despite disliking opioid-associated sluggishness, fatigue, constipation, and physical dependence. She expressed ambivalence about opioids, noting that if she could use them without unwanted effects, she might still occasionally use them and use kratom for *“coming down off of the opioids.”* She noted that when she was using both opioids and kratom: “*I would use the pain meds, but the two—you can’t use kratom and the pain meds at the same time cause they basically cancel each other out. Yeah, it’s something to do with the, the way they attach to the opioid receptors. I mean, I would probably wait at least 2–3 h, you know, if I was using a pain medication before trying kratom, and I used *[kratom]* when I would run out of pain medication; it definitely helped with any kind of withdrawals.”*

This participant had purchased kratom from 4-5 online vendors before settling on two she found to have consistency in their products. She purchases smaller bags, noting that bulk quantities can lose potency. During her initial kratom dose-finding *“learning curve”* she experienced what she called *“the wobbles,”* an unpleasant state in which “*your eyes start to feel jittery in your head*,” which she reasoned meant the dose was *“too high.”* The “*wobbles*” led to her vomiting once or twice. It had been years since she experienced this now that she knows *“how to dose,”* her preferred method being *“toss-n-wash”* (orally ingesting a scoop of powder and chasing with liquid).

She said that her kratom effects depend on the strain (kratom is sold in “red,” “white,” and “green” “strains”, although there is no scientific indication to date that different sources or “strains” for kratom exist). She reported using a red strain at night if she needed help sleeping and a white strain during the day for energy. She did not report *“hangover effects”* from taking kratom at night. She was routine-oriented, beginning her day with a *“blend”* of strains for analgesia and energy. Her *“sweet spot”* was the second dose: *“…when I take the second dose, about 3 hours later, I usually take the white strain…and then I can, in about 40 min to an hour, I feel a surge of energy and kinda, a little bit euphoric, but, but not ‘high’…I don’t feel impaired.”* She described increased alertness with kratom, in sharp distinction from opioids (“*like day and night*”). Although she had initiated kratom primarily for analgesia, and still used it for analgesia, her current primary motivation was to boost her energy and enhance her daily life.

She reported no severe negative effects and had not tried to quit kratom, but lowered her dose over time, admitting that she had initially been too *“willy nilly”* in not measuring her doses as precisely as she does now (earlier doses were roughly triple what she was taking at the time of the study). Unwanted effects primarily included tolerance, which she managed by lowering the dose:*“… I don’t know that *[kratom]* works as good for me as it used to. Um, I don’t find that there’s any super negative--but I do feel that there’s a dependence. It’s like coffee, you know? You get used to drinking coffee. But I don’t crave it necessarily. I’m not, like, going through withdrawal if I don’t take it. I mean, I haven’t tried to stop taking it completely so there is that mindset that I do worry about that if I decided to get off the kratom, too, I would go through some withdrawal. I take a low dose compared to what I’ve seen some of what people take, I’m, like, shocked at what I see on Reddit.”*

She expressed uneasiness about traveling through states where kratom was prohibited and indicated that her spouse, retired from law enforcement, would like her to quit because he *“thinks it’s weird.”* She did not want to see kratom prohibited and would rather have regular testing of kratom imports. She feared that even that could become a *“slippery slope”* increasing costs for everyone: *“*[I]* would like it regulated if it wouldn’t put people out of business and would be the same quality.”*

She expressed complicated thoughts about the US Food and Drug Administration (FDA), scheduling by the Drug Enforcement Agency (DEA), and the pharmaceutical industry; she ultimately advocated for more scientific study of kratom, but without replacement of the kratom industry by the pharmaceutical industry. She felt the two could coexist: *“…it’s not like they’re gonna, like the pharmaceutical companies is gonna take, like, the alkaloid and make something overnight. They have to do their due diligence to get it approved, you know? And that would take years and that you would hope there would be proper testing and that it would be safe. If you could have both available that would be great.”*

### 3.2 Participant 2: *“Yes, I actually used it to, um, help me stop drinking… but now that I’m not drinking…it’s just for, like, the energy, the joy of movement.”*

Participant 2 is a 26-year-old female who learned about kratom in vape shops and from TikTok videos. She had reservations but began seeking information online about possible uses of kratom for someone with a complex history of substance use and health issues: *“I have a lot of back pain. I have had sciatica for 10* *years actually. So, it’s been a long battle on that, and I also had an, uh, opioid issue over 4 years ago….and since being clean off that, starting *[kratom]* was a little worrisome.”*

Her initiation and continued use of kratom was also complicated. Although a daily, heavy cannabis user, she considered herself in recovery from both opioid use disorder (OUD) and alcohol use disorder (AUD). She had used kratom initially to address AUD (as she was unable to access inpatient treatment) but had also used kratom for pain while still drinking. Motivations in her now second year of kratom use included perceived benefits for her attention deficit hyperactivity disorder (ADHD) and her depression (supplementing prescribed antidepressants). Her kratom use is now primarily driven by *“…pain relief, and energy and mood uplifting *[effects]* to be able to get through the day.”*

She purchased about 75% of her kratom online and the rest in vape shops. She took kratom with caffeine, citrus, black seed oil, magnesium, nicotine, and cannabis, depending on the strain, time of day, and activity:*“…white is for flight, green is for in between, red is for bed. That’s how I could learn the strains….It’s weird cause, like, some strains of it, you can use like Adderall, actually. There’s some white strains I prefer when I was in my *[alcohol]* issues, I liked downers more than others. So, I have ADHD myself, so I’m like, ‘let me try a white strain’…I was happy I could focus (laughs) but I was like ‘that’s really weird that the same thing can make me so hyper and jittery but then knock my butt out…it’s a very funny plant.”* Despite comparing kratom to Adderall, she also noted: *“… kratom is more equivalent *[to]* like an opiate, and then kava is kind of similar to *[a]* drinking *[alcohol]* type-of-plant.”*

She described a 1-month “dose-finding,” having to *“…play around because you have to find your sweet spot for your dose.”* During this time, she felt she twice took “too much,” on one occasion accidentally taking two 5-g doses within an hour: *“Like, literally the worst thing that happens if you accidentally take too much kratom, you get sick…like you’re so drunk, like drunk goggles, the room is spinning, you feel like your head is--lightheaded, you feel like your ears are clogged, kind of burst situation and you’re so dizzy. Sometimes your vision can go a little weird (laughs)…The wobbles, it’s like I don’t wanna say, like, ‘overdose’ but it’s ‘you overdid it.’”* Taking too much of a white strain was comparable to taking too much of a stimulant: *“Apparently, I took too much that one time, the first time I ever did it…I felt like I took too much Adderall. I was all spazzy, shaky-like.”*

She reported side effects, including restless legs and night sweats, feeling sweaty and flushed occasionally after using, and fatigue when she *“come(s) down”* from her last dose. She has lowered her dose over time, with her highest daily dose being 30 g for optimal effects: *“…less is more with this one. The less you take, the more effect, like better euphoric, or if you take too much it could make you get the wobbles.”*

She noted tolerance as the greatest unwanted effect: *“…I started increasing my doses by accident because I was starting to get used to it. And then you don’t feel the effects anymore. *[The tolerance]* builds pretty quickly and, I’m gonna say like sometimes if I don’t switch *[strains]* up all the time, it’ll be easily like, it’ll happen in a week or two…if you switch the types of strains and vendors as well that kind of helps avoid that. Like, I normally do a mix of at least two types of kratom.”* She did not take “tolerance breaks” but rather tapers—periodically lowering her dose. She acknowledged *“…some dependency, so that’s why I taper versus take tolerance breaks.”* She considered her cannabis dependence *“way worse”* than kratom dependence.

She emphasized the importance of reasons for use: *“it really is all intention. What you plan to do and what your reasoning is behind it. Like, yeah, there are some ways to get recreational (with kratom), but honestly it, in my opinion, falls on same scale close to where more marijuana is. More as like of a risk factor. Like, I don’t think people are going to overdose on it….if you abuse it, it’s gonna abuse you.”*

She also discussed kratom withdrawal symptoms and the potential for kratom for self-taper from opioids: *“I’ve told people about *[kratom]* dependence and withdrawal possibly. Like, just because I’m saying it’s not as bad as an opiate, but I will admit it is, it’s uncomfortable….If somebody’s had a previous opiate issue, I would say *[kratom]* is a little bit of both *[a risk and helpful]*…Now, if you’re going to do somebody who is trying to transfer from Suboxone to kratom, that’s different [than from heroin]…that doesn’t, to me, fall under that category because the whole point is you’re trying to get through *[Suboxone]* withdrawals and you can taper better, because withdrawals from kratom are not anything compared to Suboxone. If you’re not in an area where you feel like you have self-control—because there’s some people who have very addictive personality-they’re gonna consistently try and get that again. If you’ve better control, then you’re able to control the kratom responsibly.”*

Participant 2 did not have recommendations for the kratom industry other than making the products less expensive and labeled clearly when mixed with cannabinoids. Discussing kratom extracts, she noted: *“I’ve been wanting to look into *[extracts]* a little bit. I got some from a vendor that sold small things for, like, this certain type of different, like, powder extract or isolate or something…they’re very potent. Like that’s the one thing, if you’re gonna do recreational, you grab those.”*

### 3.3 Participant 3: *“I would probably do the things I want to do without kratom …but it just helps me, it helps my quality of life…my life wouldn’t be destroyed by not taking kratom…it’s a performance enhancer, I would say, definitely.”*

Participant 3 is a 45-year-old male who learned about kratom from his wife, who is *“really into herbal medicine.”* She recommended he try kratom after athletic workouts. He noticed benefits: *“I do a lot of training, I do triathlons. I do a lot of running, biking, swimming, lift weights, so I have body aches and soreness; it’s just, I’m getting older now, so I just feel sore sometimes…And it’s been 6 years and, to date, I don’t have an experience of anything negative from my use and I’ve not increased my use or anything.”*

He had decreased his use over time, tailoring it to his activities: *“…it was experimenting, when I first started, I think I took too much…only the tea when I first started, I think it was a tablespoon…but now, you know, I can toss-n-wash and it’s a teaspoon, which I think it’s between 2–3* *g, maybe…I take it a lot when I’m actually exercising.”* During training or a triathlon, he would take 4 capsules halfway through.

He described both analgesic and stimulatory effects; when asked whether they were simultaneous, he responded: *“Oh, definitely. Yeah, I mean, even today I wasn’t groggy *[prior to taking his dose for the study]* but you still feel it, it was almost like you drank a little bit of coffee or something *[post-dose]*.”* He also reported mixing kratom with coffee and enjoying the taste of kratom.

He noted a lack of euphoric effects from kratom: *“I was talking to (my wife) the other day about it--I’m not on the forum as much as I used to be but--and I was like “you people must be taking something different than what I’m taking because I never experienced euphoria” … cause I had Oxy, after I had my knee surgery, and I was like, “I’ve never felt anything like that from kratom,’ and *[my wife’s]* like, ‘I have a couple of times, I’ve felt that euphoria.’ I was like, “I’ve never felt that euphoria’… It hits her differently than it hits me… I’ve never felt like euphoria from it. I’ve never felt like I was on a drug, like an intoxicant.”*

He currently purchased kratom from 2-3 online vendors after rotating several. Like other interviewees, he felt that “less is more” and was puzzled by high-dose use: *“Less is more is kind of the approach I take, and I don’t, I don’t see how some of these people can do what they want. I just don’t understand some of these grams that people are throwing down. I don’t know what, um, some of the other people in this experiment are doing but, um, you know you’re hearing (online) like 20, 30, 40 g that’s, that’s a lot. I don’t know. I don’t even know how you would consume it.”*

He reported one adverse experience from kratom: *“…when I first started off and messing around with dosage I definitely probably took too much, got sick, and threw up, yeah, and I had the shakes like this is all early use…my eyes would like kind of like twitch a little…this was a long time ago…I was experimenting with the doses at first and I took too much and like it was called ‘the wobbles’… I don’t even know how to describe it. Like, you just felt like your eyes were like wobbling; it just felt like you were just kind of, like you were out of it. It wasn’t like I needed the emergency room, it was just like, ‘this is weird’ and I read about it, like, you get the wobbles if you take too much…I felt weird for like 15–20 min, I took a shower and it kind of went away.”* The only long-term risk that concerned him involved his *“liver enzymes,”* but he said that if he were to feel an adverse effect from kratom, *“it would have happened already.”*

He has taken 7–13-day tolerance breaks but has never tried quitting as he felt no need to. His tolerance breaks did not lead to withdrawal symptoms except as follows: *“I would get restless legs at night when I would go to sleep, but I wouldn’t feel anything during the day. But that hasn’t happened every time.”* He reported trying to take 1–2 days off from use per week. Although he rotated strains to reduce the likelihood of tolerance, he expressed doubt about strain differences: *“I think it’s psychological. I know the reds are supposed to be sedative and the whites are supposed to be energetic and the green supposed to be in between, but I don’t know. They kind of all…feel about the same.”*

Like at least one other in this sample, he had spoken about his kratom experiences publicly, but he became less open about his use due to his career and volunteer work. He expressed concern about rhetoric from kratom advocates and opponents: *“Obviously, I don’t know, kratom or something is causing problems in some people. So, I don’t know what I would really say other than that for me, kratom works fine-I’ve had experiences, there has been no ill effects—so, it’s hard for me to understand why there is this much interest in kratom. It, it still baffles me. So, I guess let the research play out and prove that what I kind of already know. But, I think to resist--I think, who was the podcast I was listening to--but it was basically like when they talk about banning kratom, it’s almost like saying you’re gonna ban booze and not distinguishing between beer and liquor.”*

He nonetheless described need for change in the industry (calling the market *“just weird”*) and expressed worry over extracts: *“There definitely should be regulation. I want to make sure that the package I get has kratom in it and nothing else… I don’t know what to do with the extracts cause it works for people; on extracts there should be a warning label or something. But I think the extracts is what’s causing all the problems as far as overdosing or whatever is happening… or paying several hundred dollars a week on kratom, they’re spending several hundred dollars a week on extracts, I guess.”*

On the American Kratom Association (AKA), he noted: *“The new leadership seems more focused on fundraising and, uh, some of their tactics, I didn’t really agree with…I worry that they’re going to squeeze out a lot of people, they’re going to make it so that they’re kind of the novelty of the kratom world where vendors can only, even with this Kratom Consumer Protection Act, they’re almost making it so if you’re not an AKA-approved vendor, then you can’t operate in the world. That scares me a little bit. But, I mean, they serve a purpose… I liked it better when it was more grassroots, this feels more like Astroturf now.”*

### 3.4 Participant 4: *“Like I always told my clients, you know if it works, keep doing it, and if it doesn’t, well, there you go.*”

Participant 4 is a 49-year-old male who learned about kratom from clients when he was a counselor at an OUD clinic: *“…the (clients) had told me there that people use it to get off heroin. So that’s kind of what I got curious…so, I went out and I realized that, well, (kratom’s) probably more benign than the media has said, so I got a little pack and yeah, well, I’ll try it.”* Having no OUD history, but not being opioid naïve and wanting not to encounter anything as strong as heroin, he said he tried kratom from curiosity and because clients had described kratom like *“a frozen pizza with nothing on it but cheese”* whereas heroin was *“a supreme pizza.”* He expressed irritation with sensationalist claims: *“Everywhere you read on the internet or looked it was, like, toxic. You’re gonna die, it’s worse than heroin.”*

Although he began out of curiosity, he continued use to treat stress, sleep issues, anxiety, depression, and treatment-resistant Irritable Bowel Syndrome (IBS) gastrointestinal-specific (more so than pain) symptoms. IBS and mood and energy improvement were his primary reasons for continued use. He described early effects from low-dose experimentation as somewhat paradoxical: *“…you get that warming effect, even like for someone who’s never done it. And when you use it first, you get that. But you also get that burst of energy, you feel good, a little bit hyper even.”*

He purchased kratom online from vendors who appeared reputable, based on adherence to Good Manufacturing Practice (GMP) and GMP-certification, noting that they claim to test for contaminants. He used different strains but acknowledged that: **[vendors]* put a lot of labels on different strains, but there’s really just a couple…they’re just marketing it.*” He noted that the products don’t always perform as labeled.

He had experienced side effects from kratom and was unable to take it on an empty stomach. He thinks kratom can be *“an appetite suppressant”* at higher doses and finds this *“annoying.”* He eats within 15 minutes post-dosing for the 2-3 doses he spaces over the day. Another side effect has been irritability: *“I was diagnosed with bipolar and there will be times when I feel bipolar. I don’t think It’s bipolar. I think it could be the kratom tweaking me a little bit. Edgy, a little hypersexualized. It’s very random… it just comes out of nowhere once or twice a week. It seems like the white strain is making me an irritable motherfucker…I noticed that if I take white in the afternoon or evening I get a little cranky sometimes.”*

He said that when he takes too much (for him 4.5 g), he feels restless and has difficulty sleeping. When he previously only used recreationally on weekends, he once tried an extract and then, later in the evening 5 g of powder. When asked if this had resulted in “the wobbles” he responded: *“I did not get them, but I’ve heard of that. I would say that I was probably close. I was floating like, uhm, I just remember, like, those cartoons when someone gets clobbered, they see those little things, birdies going around. I did feel like that.”*

With respect to extracts, which he has used only occasionally, he said *“…there’s the possibility to get your organs a little fried up there.”* With respect to higher doses generally: *“I guess there could be some harm in that. I think the powder… your body is gonna reject it. I don’t believe in any sense that, like, your body can handle *[a lot of]* powder. But then I’ve seen, I was telling *[name]* this, I’ve seen, uh, when I was in some of these Facebook kratom groups…some people, the doses they’re taking is…and, you know, Reddit, it’s absurd! How do you not throw up?…You just don’t need that much.”*

He described instances where his dosing had become too high (5.5–6 g three times daily) and impacted him negatively. He had quit but resumed use due to life stressors: *“I had days of feeling like actual ass. I was done… I said “I am never touching this stuff again.” I was just taking too much, and it wasn’t really helping. It was making me feel worse…I had a cup, that you know, I used to put my kratom in all the time. It was deep green. And I had a scale--I don’t like eyeballing it--and I threw the scale away…. And then, my dad died, and my mom died. I had to get surgery on my kidneys *(*unrelated to kratom use*)*. *(*Then*)* I was, like, cruising the mitragynine website, ‘yeah, I feel like shit, I hate myself right now, this sucks ass,’ so I started using again.”* He had experienced depression after quitting, but noted it had coincided with seasonal depression and life events. He said he would never again quit cold turkey.

He took tolerance breaks by tapering but not quitting completely (because quitting seemed to worsen his IBS symptoms). During tapers, he had withdrawal symptoms that partly contrasted with those from caffeine: *“I can go a day without coffee. Like, I was falling asleep in there, it’s because I didn’t have coffee. I get a headache; already passed through the headache, and then, then the depression kicks in. But when you pull away from kratom you, if you don’t taper the same way, you’re gonna go through a similar thing but not depression. It’s gonna be more anxiety, more restlessness…the last time I went off of it, uhm, I just tapered off of it for a couple days and then I was fine.”*

He said he is open about his use and rebuts what he considers misinformation. He differentiated between recreational and medicinal use of substances, saying that there is room for both and that kratom can be used “medically”: *“There is a lot of people with chronic pain issues, IBS, doctors that do not want to help with, or cannot help with… I am getting on my high horse here: I believe it is people’s right to have access to this. If it is regulated, fine, if it is 21 over, fine, if it is good consumer manufactured, that is fine. But the claims that *[media and government]* were making at certain points--and you know how FDA always jumps in there…one doctor was quoted on there, you know, like news article, ‘kratom is 100x more potent than heroin,’…we jumped all over that. The *[online]* groups I was in pointed out, they went on a rant about how insane it was.”* He ultimately wanted kratom to stay legal, but with high industry standards for *“safety issues.”* He expressed suspicion that government could be complicit in profiteering from kratom.

### 3.5 Participant 5: *“But, yeah, as long as I take those breaks, it still is helping my anxiety, you know, just like it did back when I first started taking it.”*

Participant 5 is a 35-year-old female who learned about kratom doing online searches for natural medicines for anxiety and depression. She had been prescribed antidepressants and anxiolytics, each with unwanted side effects. She had also tried cannabis for these issues, but reported it caused impairment to the points she could not function. Concerned about physical dependence on prescribed medications, she had discontinued them: *“I did, like, a Google search for like natural---I went through some stuff like, there was kava, and I tried that, and it didn’t really do anything for me. CBD, and CBD never really done much for me…Kratom, popped up and I said ‘you know, I’ll give it a try.’ I ordered some and, you know, after taking a few times and kind of finding what worked for me…I liked that it took my anxiety away and it (was) something actually helped me. And it wasn’t a prescription.”*

She learned that she preferred kratom in capsules, disliking the taste of raw powder. She said she continues using primarily for anxiolysis and energy: *“… it’s just been very consistent with--you know, I get up in the morning and it’s almost like a cup of coffee. I take some and it just, you know, like, my whole life, I’ve dealt with bad, bad anxiety and depression, and, you know, I get up in the morning and just worry about everything and just stress myself out to the point I’m sick… I don’t feel that way anymore. I take it and, you know, it’s, it just mellows me out a little bit so I can get through the day.”* She said she also occasionally uses to alleviate minor aches, including extract use after a back injury.

She said she doses 2–3 times per day, typically at the same times. She elaborated on kratom’s paradoxical effects: *“It is very hard to describe, like, it does give me a boost of energy but at the same time calms me down… kratom is uplifting in a way where it can, be a mood booster. It wouldn’t necessarily get you high. It doesn’t get you high, in my opinion, it just puts you in a better mood. It kind of uplifts me and it’s almost like drinking a cup of coffee…at the same time I feel my anxiety, you know, mellows me out…It’s hard to describe because it’s kind of counter, but it works.”*

She described managing tolerance with 5–7-day breaks 2–3 times yearly: *“…I just kind of taper off, take a little bit of a break and then I take it again and the effects are stronger.”* She has never attempted to quit, but during the tolerance breaks described: *“I don’t really get the bad withdrawal that everybody says. I’ll notice the little things like I’ll get a little achy, a little bit of a runny nose.”* She also noted restless legs during tapers, though she has a history of that. She summarized withdrawal: *“…it’s not anything I can’t get through. It’s not unbearable. It’s just…I’m not feeling 100% top-notch, but it’s not unbearable. I can get through the day.”*

She noted that *“the littlest things can make (kratom) not work”* and that she must take kratom on an empty stomach to experience effects. She also described unwanted effects, including constipation she manages with fiber pills. During her dose-finding she experienced adverse effects after taking too much:*“…at the beginning when I was, you know, kind of experimenting—and I’ve learned that different vendors, too, like some of their kratom might be more potent than others…I took too much…I just felt really nauseated and, like, I had a real bad headache and I had to lay down. I felt dizzy…that’s the extent to what happens; you won’t overdose, you’ll just feel really sick…I just drank a lot of water, and I laid down and just slept it off and I woke up and felt a lot better.”*

She said she purchases capsules online from 6-8 trusted vendors. She expressed wariness about extracts: *“I’ve seen a lot of these gas stations and smoke shops selling these extracts…somebody that doesn’t have much knowledge, they’re just gonna go for that. And… maybe get hooked on that and, uhm, I think it’s just something that, you know, if you use it once in a great while…I might get an extract shot or, um, extract tabs or something once in a blue moon.”*

She was skeptical of kratom being developed into a pharmaceutical, saying that she would *“rather stick with the vendors”* than take an FDA-approved medication derived from kratom. Still, she noted that the industry could self-regulate better: “*When you get a lot of these pop-up vendors that just decided to sell and, uhm, like for instance a lot of these vendors now they’re legit they’re getting kratom laboratory tested and they have, they can provide anybody with a lab test saying this is good, this is the levels of everything. And then when you get a pop-up vendor and they don’t have anything of that, or have a clean workspace, I think that’s what gives everybody a bad rap… they need to be more consistent with how these other vendors handle things, with getting their laboratory tested…show a clean workspace--I think it makes it look better when you do that versus a pop-up; you’re doing it out of your house and Ziploc baggies. They’re kind of, you know, a little questionable.”*

Ultimately, she emphasized benefits from kratom with little downside: *“…I take it every day and I drive, go to work, you know. And I’ve taken it now since 2016. I’ve yet to go to get in any accidents. I’ve, um, excelled at my job…It's just it's a supplement to take to help you get through the day and, um, I’ve never felt, you know, impaired at all on it.”*

### 3.6 Participant 6: *“…kratom definitely helped me, like, during the second half of grad school in terms of, like, putting your nose to the grindstone and finishing your thesis…”*

Participant 6 is a 34-year-old male who learned about kratom on Reddit. He said he enjoyed trying new drugs, but did not identify as a “psychonaut.” He first purchased kratom in a head shop, but then purchased online. He began using kratom when he was a doctoral student in a small town, feeling bored and isolated; those feelings, and curiosity, helped motivate his use. He was using cannabis, poppy seed tea, and had previously taken prescription opioids *“if they’re there.”* He had briefly experimented with psychedelics and with other psychoactives marketed as “research chemicals.” He was intrigued by kratom’s effects and described his first 5–6-g dose: *“I was like, ‘oh, this is similar to poppy seed tea’ in that it’s somewhat relaxing and dreamy, but also, it’s, like, similar to coffee in that I don’t want to just pass out…like, you could do things on kratom.”*

Kratom’s availability and price inclined him to keep using. Over time he noticed that kratom *“helped somewhat in terms of, like, focus and reading, and writing was a big thing.”* In recalling how his use motivations had changed, he reflected: *“I’d say like in the grand scheme of things, I started recreational and then sort of migrated towards using it as a hybrid-like recreation-and-productivity in one…I didn’t try kratom thinking like ‘oh, this is a study drug’ and I didn’t, like, start using kratom for the purpose of doing work, umm, until, like, probably a few months or a year after I like very first started.”*

Other benefits included energy for exercise and socializing after work: “*I’d, like, finish up the day in lab, take some kratom, and then be ready and raring to go do more social things after rather than just crash, yeah, or like “veg”… kratom helped with that….I was using kratom as, like, a pre- and post-workout supplement, where, like… it did motivate me and kind of helped with the aftereffects of, like, going to the gym.”* He summarized effects distinctly: *“…like Vicodin plus coffee; like, two Vicodin plus a redeye is what you’ll feel from kratom. It’s like the feeling, kind of this mix of, like, energetic, get up and go, plus like, dreamy, euphoria. I don’t even know if I would use, like, “‘euphoria.’”*

Unwanted initial effects included increased appetite, particularly at higher doses, but he differentiated this from cannabis-induced *“munchies.”* He reported constipation, successfully managed with fluids and fiber. He also noted increased warmth and perspiration after using, along with irritability: *“…at times it didn’t help with, like, my temperament… I had a much shorter fuse with kratom.”* He suspected that this occurred more with white strains and *“certain batches.”* Another unwanted effect was sexual: initially kratom provided*“…longer performance, stamina, or, energy-wise, like, delayed everything. Um, nowadays it’s more like I’m just less interested in that in general…not great.”*

This participant described an occasion when he took too much: *“…it was early on for sure, it was probably, like, uh, 8-g dose…I wouldn’t say double what I started with, but definitely higher—and, uh, like I would get, like, sort of weird tunnel vision, like cold sweat situation, uh, shakiness…I would have to sit down for sure. But it only happened, like, bad, maybe once or twice…it was like an hour tops.”* When asked if he thought kratom overdose is possible, he responded: “*I think people can, like, take too much--I don’t think it would ever, like, kill you, like I never felt I wanted to…like I was about to die on kratom, but, like, there were times when I ate too many Vicodin and I was, like, ‘I think I’m gonna, like, not pan out well.’”*

When he used consistently, he used between 10–15 g per day. He described having been initially more cavalier with dosing: *“I probably like fluctuated with dosage, um, never with the intention of quitting per se. It used to be, like, I would take like 20 g in a full day willy nilly. Like, whenever I had a chance, I would eat like a few grams. Nowadays it’s more regimented…in the morning I’ll take this amount and then in the afternoon I’ll take that amount and then that’s it.”*

On physical dependence: *“I wouldn’t say it’s an issue, but I definitely did get tolerant to kratom in the sense, like, when I very first like started like a 5-g dose would, like, almost made me puke. Whereas, like, months later a 5-g dose would--I’d still feel the effects from it, but… I wouldn’t want to vomit it up…even as I tapered down, lowered my dose more recently, like, I didn’t feel like I was withdrawing or anything, um, the effects were just less.”*

He was actively tapering his dose in order to quit, because his girlfriend did not approve of his use and because his recent lab panels had shown elevated liver enzymes. He rarely drank alcohol, and he suspected a contribution from kratom.

Despite benefits he described over the years, he cautioned*: “I would also warn people more now that, like, it is insidious in the sense that… there’s a lot we don’t know about it. It’s literally like being harvested in the jungle, like, drying on the jungle floor, getting shipped here and then you eat it.”* When asked whether he had ever been addicted: *“I’d say that I was addicted to it in the sense that… if I don’t have it, I feel wrong, like, it’s not a normal—I feel like, like something is missing…so, I wouldn’t say, like, I’m addicted to it in the sense of, like, I’m seeking to use it all the time, but I am aware that when I don’t use it, I don’t feel right. I don’t think it’s, like, affected my life in a negative way to an extent where it’s like, ‘oh, it’s a real addiction.’”* When asked to define “real addiction” he clarified: *“It’s not preventing me from, like, achieving certain goals.”*

### 3.7 Participant 7: *“…it’s not a cure-all, it’s not a panacea, it’s not, you know, something that’s gonna give you your old life back, but it can make you very much more comfortable.*

Participant 7 is a 52-year-old male who learned about kratom from a friend who had chronic pain and who had a daughter *“battling a heroin addiction”* who had used kratom to attenuate heroin withdrawal. He looked up kratom in online sources, including pain forums with older adults, and found that it seemed *“legit”: “I didn’t really do anything about it, and then…I was at the right gas station, and there’s a whole rack of it in there. I thought ‘What the hell? I’ll try this stuff!’ It was kinda love at first bite. (laughs).*”

His initial reasons for trying were twofold: severe, chronic pain (with concomitant sleeping disturbances) from an 8-year-old construction accident, and wanting to abstain from opioids: *“I had an issue with pain pills…I played football in college in the ’80s, and here they handed them out like candy. I got my first (oxycodone product) when I was 14* *years old…we didn’t know the dangers of it, but it got to the point where, you know…I would get a prescription…and that would last me maybe 3* *days…I really got dependent on them. I got clean without the kratom. I realized there was a problem there, you know? I actually went to rehab…went to a sober living house for a while because…I gave up control of it…I tried everything.”*

Following his injury and surgeries, he always *“over-took the pain medicine.”* He expressed frustration that he reached a point where he could not continue using opioids, but still needed pain management—his primary reason for continued kratom use: *“… it’s definitely not an opiate, but it’s way better than ibuprofen…it’s not so much ‘peaks and valleys.’ The opioids it seems like, you know, they come on and they hit you and you get the euphoric rush and then it’s all downhill… after, I’d say, an hour or two, after taking the opiates, the beneficial part was over. It was all the detrimental part. It was the itching, it was the sweating, it was the craving for more. With this, it’s kind of smoother. It’s not as high, but it’s also not as low…I always wanted more *[opioids]*; with kratom, if I take too much, ugh, it gives me kind of a ‘blah’ feeling, it’s kind of a negative return on investment, I guess you’d say. I don’t know, it’s not like I feel like I could overdo it.”*

Describing a time when he took too much kratom during his dose-finding phase: *“…it’s like when you do cocaine and you have all the energy in the world, but you can’t get out of your chair, you know? It’s like this restlessness but inability to act on it.”* During that dose-finding, when he used 4 times per day, he had experienced perspiration and difficulty sleeping. Now, using less, he said he has no unwanted effects (but dislikes kratom’s taste).

Although he used primarily for analgesia, he noted other benefits: *“…it kind of helps my focus you know?… it seems like I’m a little more on task and a little more, you know, productive throughout the day with *[kratom]*.”* He elaborated that kratom helps him work or exercise at modest doses: *“Less IS more. You know? And if I just need an energy boost, I’ll take one scoop instead of two, you know? If I’m just feeling a little sluggish and want to go to the gym. When I have pain, I’ll take two, but if I just need a little bit of energy I’ll take one and it works way better.”*

When asked whether kratom helped him resist temptations to return to opioid use: *“Oh, for sure. No, this is something that’s legal, and it’s something that… I don’t feel there’s an element of danger to it, I mean… that’s why I want to participate in the study, because if you guys find out that it turns your liver purple or something… [laughs] you know, I’d like to know that…as far as I’m concerned, it’s just kind of a juiced-up version of coffee… I mean, it’s a step above, like I said, it’s not an opiate, but it’s not ibuprofen.”*

Although he never attempted quitting, he discontinued use for 3 weeks when moving. Withdrawal symptoms did not begin until 3 days after his last dose. These included restless legs, muscle clenching/spasms, cold sweats, and irritability. Comparing kratom withdrawal to opioid withdrawal, he responded: *“(laughs) not even close. A walk in the park”*

He was the only participant to describe encountering adulteration: he had tested positive for opioids on a urine drug screen for work after having used only kratom products. This incident, along with other kratom marketing trends and lack of standardization, disturbed him: *“Turn off the hype machine. All the BS with the different strains… and this and that, the vape pen, and whatever kind of BS. Legitimize your product. Standardize dosages. Make it easy for people to figure out…I know there’s charts that they say, you know, 2–4* *g for this, 4-6 for that, 6-8 for this. Come on… just try to do your best to put on a standardized product, and sell it for what it is, you know?!?…Once the marketing people get into it, you know, then you have *[kratom]* gummies, and you have all this stuff…have ya tried a gummy?! They’re awful!”*

### 3.8 Participant 8: *“…it never, like, hinders my ability to do things; it may make me more willing to do things that I might not feel like doing before.”*

Participant 8 is a 32-year-old male who learned about kratom on Erowid at age 15: *“I used to frequent [Erowid] a lot when I was younger, just to learn about the things that I wanted to try and experiment with, and I remember seeing (kratom) on the front page…I hadn’t heard about it, so I clicked on it and read about it…it just like sat in the back of my mind… but I didn’t try it until I was maybe like 18, so 2008. That was just a period when I just started pretty much experimenting with whatever I could.”*

Despite drug experimentation, he did not consider himself a “psychonaut.” He reported purchasing kratom online once, but not using again for several years. His later roommate regularly used kratom and he then began to *“explore it a little bit more*.” His primary motivations were curiosity and recreation: *“I wanted to try something new…it sounded like something that I would enjoy the effects of…at that point in my life, like being 18, like I wanna try different drugs and stuff—that and the fact that it was legal and easily available, and I was able to get it online… it sounded interesting, it didn’t sound like a harmful thing, and it was easy to obtain so I just ordered some and figured, ‘why not?’”*

He began regularly using 4–5 years ago and has used daily for 3 years. He described benefits in mood and productivity, but highlighted kratom’s pleasant effects as additional motivators: *“…it gives me energy for sure…like, motivation, and just cause it makes me feel good, you know?…It, it gives me, like a euphoria, that feels like typical of, like, an opiate, like what most people get from them…I’m just in a better mood generally around people, more talkative, more energy.”* He also uses modafinil daily for productivity. When he was without his modafinil prescription, he used more kratom to substitute.

Asked whether kratom impaired him: *“No. When I use it, it more enhances my, like, functioning, rather than impair it…like cognitively I can focus better…I can get more into whatever it is that I’m doing, and then, you know, the highness and euphoria also helps drive that feeling to keep doing whatever it is that I’m into.”*

He developed tolerance since starting to use twice daily; he has not undertaken tolerance breaks, but said he sometimes uses only once daily to reduce tolerance. He noticed tolerance forming at a 5-g dose. When he increased to 7–8-g, he recognized that he should likely reduce, but thus far has not *“made much of an effort,*” believing he can satisfactorily maintain his current regimen by switching vendors and strains. He had no plans to increase his dose or to use extracts regularly because doing so, he acknowledged, would increase tolerance. He never tried quitting because: *“I’ve never felt that my use is really causing any issues, at least not as of now. Like, if I start taking a lot more anytime soon, then that might be like a sign to me. But I think that with my current usage habit it’s improving my wellbeing.”* Indeed, he remains open about his use, explaining kratom to coworkers who ask about his *“green coffee.”*

The only side effect he noted, besides tolerance, was mild constipation. He had experienced one adverse event similar to what others called “the wobbles”: *“I wouldn’t even have to take like a really high dose, even if I just took like a normal dose—I guess it’s like nystagmus, like kind of like wiggly eyes; it’s hard to explain it. Like, maybe if I looked off to the side, my eyes wouldn’t really stay steady, and that would kind of cause, like, a vertigo-y feeling. But I’ve only noticed that from this like one particular strain from this one person.”* With respect to overdose: *“I feel like people can take too much and get uncomfortable and have a negative experience, but as far as like ‘overdose’ to the point where it would like drastically affect their health, I don’t necessarily think that it could happen with kratom alone… from what I’ve read, I don’t believe that kratom alone can cause enough respiratory depression to be fatal…those cases are always in combination with other drugs or opiates.*”

Unwanted effects occurred: *“…when I’ve taken too much without a lot of food in my stomach…within an hour, ended up throwing it up, and it’s just never really a fun feeling.”* He speculated how he would feel if he were to dose too much: *“I could probably handle it, but when I get into like 9–10* *g, that’s the point where I might get some nausea, or I might be more sedated than alert, and that’s not really what I’m looking for when I want to do kratom…the strains that I tend to like are whites and greens, because I like the energy boost.”*

Like others, he wanted industry change: “*Transparency of knowing exactly what’s in it…where it was harvested from—sometimes strain names can be a little ambiguous or misleading; sometimes I feel like people are just making them up…just taking the same thing and putting it into three different bags, and there’s no real standard, or way to know. If it was possible to see, like, alkaloid composition, that would be really neat, too.”*

### 3.9 Participant 9: *“… I drink green juice every day, I take a stack of supplements that I cycle through, y’know? So, it’s just like another supplement to me.”*

Participant 9 is a 41-year-old female who learned about kratom from a friend who recommended it to her when she was in pain from undiagnosed Lyme disease. She had no reservations about trying: *“I’m a supplement junkie. So, first of all, I didn’t look at it as a drug… I definitely did my share of drugs when I was younger, too—not, like, addictively, but—I’ve never been one to be, like, ‘Ooo! Better not!’”*

She described disliking both smoked cannabis and kava and currently was not drinking after prior periods of heavy drinking. She had prior physical dependence on, but not addiction to, prescribed opioids following a car accident. She said she uses CBD and 4 milligrams of THC in dropper form nightly. Although she had initially taken kratom for Lyme disease symptoms, she found that it helped her cope with a confluence of other severe stressors (violent assault, hurricane, car accident): *“…that all happened, like, in a really short amount of time. I was, like, catatonic, for a while, and I really wholeheartedly believe that kratom helped me get my life back to: ‘OK, I can dust myself off now and go back to work, and then eventually go back to school,’ and I know I wouldn’t be in school--I mean, I have a 4.0 GPA… it’s helped me”* She said that her primary daily drivers of use are *“focus and productivity,”* especially for graduate-school work. She said she also uses to address ADD/ADHD (reporting that she cannot tolerate prescription stimulants) and, to a lesser extent, to manage “*some depression*” and social anxiety.

Like others, she noted: *“… it works better at lower doses… So, when I get higher doses, the higher I get (in dose), the less it works, but I just keep doing it because of my personality (laughs).”* She described a complicated relationship with kratom: *“I would’ve definitely just kept it at that 2-g dose, and just kept going with taking, at least, like, a day or two off every week, so it was more of a tool than a crutch… at this point, I have a strong physical dependence.”* Her session day with us, she said, was the longest she had gone without using, and she had felt *“…pretty antsy. It wasn’t like heroin withdrawal, but y’know.”*

She said kratom was not what she considered “*intoxicating*” in the powder form she used. She described the effects as *“…very finicky…you take the same amount and it’s gonna be different every single day… if I take too much, I get nauseous. But that’s taking a lot at this point.”* She had avoided constipation by fastidious hydrating but reported getting flushed after dosing. She had also experienced “the wobbles”: “*The closest way I can describe it is that if you’re looking at something and you have, like, double vision, almost—so you’re seeing—it’s not going like that, but, like, you’re just kind of a little out of focus.”*

She expressed concern over unknown long-term effects and contaminants. She said she sticks with smaller vendors she trusts, and she called for greater industry transparency: *“I’d like to see published lab results on every bag… I’ve had vendors I, like, dabbled with, and that I realized that their lab reports, every one of them said the exact same thing. So now I’m not really trusting that you test everything that comes through your facility….I wanna know what I’m getting; I wanna know that it doesn’t have molds and metals and any other toxins. I was reading a paper that some of it has tested positive for fentanyl, and that’s terrifying.”*

Despite having felt “antsy” on her session day before taking her regular dose, she said that when she had quit once, it had been unexpectedly easy: “*The problem *(*with quitting*)* was it was so easy. Like, I thought it was gonna be this whole thing… I was geared up for it, and I had this arsenal of supplements, and I just was going about my life, like, literally the next day. So, I only made it, like, a month, because I was like, ‘Obviously I can quit anytime’.”*

She said she plans to continue using while in school, but as she begins her career will use only on weekends. Asked if she thought she could moderate after years of daily use, she had confidence: *“I’ll tell you, the only reason—left to my own devices? Absolutely not, because I’m just the queen of justification. But I have this magical box that I can put stuff in, and it locks it for up to 11 days…it’s like a lockbox that is on a timer. So, when the ‘good intention’ me that’s like, ‘OK, I’m gonna stick to this, and not touch it again til next week’--once I make that decision, I can’t break it (laughs). So that has been very helpful for all of my vices. I have currently, like, a stash of dark chocolate in there.”*

### 3.10 Participant 10: “It’s just a plant.”

Participant 10 is a 38-year-old male who learned about kratom online in 2006, exploring it because he has *“an open mind”* about trying new things. His initial motivation was *“trying to get high”* and he found he liked how kratom made him feel. When asked why he had continued using, he listed multiple motivations: *“…to help me sleep… to help with anxiety. Um, I have attention deficit disorder and bipolar disorder, um, it really helps with my mood, it helps me deal with my dad; he’s a narcissist.”* He said kratom helps his bipolar disorder *“when I’m in the depressive part of it”* more than by addressing hypomania. His primary use motivation now, he said, was to *“boost my mood”* and decrease *“stress and anxiety.”*

He compared kratom’s effects to those of THC, which he believes pairs well with kratom, but added that kratom’s effects were *“unique.”* He had used stimulants only twice and (an oxycodone product) only once, limiting the comparisons he could draw across drug classes. However, he described paradoxical kratom effects: *“You kind of feel like a stimulation then at the same time you feel a little bit sleepy…not really jittery…I heard that they say that the lower dose is kinda like a stimulant, and the higher dose is kinda like a sedative. Umm, it just seems to me that the more I take the more intense it is….I do seem to notice that when I first take it, it seems like it is kind of like a stimulant…like when I took it today…I was pretty sure my blood pressure went up…if it’s a higher dose, usually, well then, at the same time you feel kind of sleepy too, it’s kind of weird.”*

It is critical to note that this participant also regularly used 30-milligram dextromethorphan pills (and medically prescribed cannabis) with kratom, dosing up to an entire 16-pill pack. His dosing was contingent on setting and activity: *“…depending on if I was home I would take more; if I was gonna try to function I would take less. I use it a lot for when we were doing the Christmas lights (at work) because that was after regular work, we would just be outside not doing anything super important, so, I could take a little bit of it to kind of help deal with the other guys.”* He elaborated: *“other times, when I would change the filters at the end of the day, umm, when I was kind of tired, and my feet are kind of aching, I would take some and then it would boost my mood, I would feel good, the achiness in my feet would go away, and I felt like I wanted to go do a bunch of work.”* He also noted kratom was beneficial for exercise and sex.

On one occasion when he took too much (approximately 4 spoonfuls), he vomited and felt dizzy for *“a couple of hours.”* He said he believes overdose on kratom is impossible because someone taking a large amount will quickly *“get sick and throw it up.”* Tolerance was the sole unwanted change he reported, though he speculated about kratom-induced hair loss. He did not think that a person could become intoxicated on kratom unless they had low tolerance and took a high dose.

He took regular tolerance breaks: *“…I have had periods where I would just take *[kratom]* all the time and then the tolerance would go up and up and up, one spoon then three spoonfuls, and then I would just stop because I didn’t want to waste all my money…I would just take a break from it and take something else or just don’t take any for a while to build my tolerance…”* The longest break was 6 months. At the time of the interview, he was taking a 30-day tolerance break from cannabis. He was open with his use around people he thought would not judge him unfavorably, and he had no plans to quit. As he has been satisfied with kratom vendors, his only feedback for the industry was: *“…lower the price?”* When asked how he would like to see the government handle kratom he wished simply that they would *“leave it alone.”*

## 4 Discussion

We used narrative interviewing without expectation of convergence or divergence among participants’ stories but encountered both. A striking takeaway from our interviews was the degree to which commonalities emerged despite participant heterogeneity, varied use motivations, and changes in dosing over time, which have been noted elsewhere (Smith et al., 2022c; Grundmann et al., 2022).

Foremost was the consistency with which participants described effects, though all were using different products and doses both between-person and within-person over time. Analgesia, energy, and mood-enhancement were prominent. Based on the self-report of these participants (all of whom had taken kratom the day of their interview), kratom’s subjective effects were consistently described comparably to a mild opioid mixed with caffeine, or even a low-dose opioid, which can produce some stimulatory effects. Acute mood enhancement, sometimes but not typically articulated as “euphoria,” was also described. These descriptions are congruent with the emerging pharmacology of kratom’s alkaloids (Obeng et al., 2019; León et al., 2021; Chen et al., 2022; Hiranita et al., 2022; Smith K. E. et al., 2023).

Also consistently described was physiological tolerance, though the presence and symptomatology of withdrawal was less well characterized and varied across participants. These reports are consistent with prior papers that have reported on the presence of kratom use disorder (KUD) criteria, assessed using DSM-5 diagnostic criteria for substance use disorder not otherwise specified, that was determined largely on the basis of physical tolerance and withdrawal rather than psychosocial impairment or risky use (Garcia-Romeu et al., 2020; Smith et al., 2022d). It is reasonable to suggest, based on these data from long-term kratom-using adults, that kratom tolerance occurs with regular use, particularly at higher doses. In subsequent analyses, we examined responses completed by these participants on a detailed survey (completed prior to our EMA study from which we recruited). KUD symptoms were assessed. As shown in Table 4 past-year KUD criteria were met by half of participants, with mild severity met by 3 (Participants 5, 8, 9), moderate met by Participant 2, and severe met by Participant 6. Here, KUD was exemplified by tolerance and withdrawal symptoms, rather than psychosocial impairments, life-role interference, or hazardous use. These symptoms are in keeping with the experiences participants shared, highlighting the reality of physical dependence, but also the compatibility of kratom use with daily life obligations, including work, school, and recreation. We think it is worth noting that physical dependence is not considered pathognomonic for a drug that is taken under medical supervision. For kratom, that option is not currently available.

**TABLE 4:** Kratom use disorder (KUD) symptoms endorsed by participants for past-year and lifetime, and KUD severity.

Participant	1	2	3	4	5	6	7	8	9	10
**Used in larger amounts and/or over a longer period than intended**	No	Yes	No	Yes, but not in past year	Yes	Yes	No	Yes	Yes, but not in past year	No
**Made at least one unsuccessful attempt to cut down or control use**	No	Yes, but not in past year	No	No	Yes, but not in past year	Yes, but not in past year	No	Yes	Yes, but not in past year	No
**Spent a great deal of time on activities necessary to get, use, or recover from effects**	No	No	No	No	No	Yes	No	No	No	No
**Experienced cravings, strong desires, or urges**	No	Yes	No	Yes, but not in past year	No	Yes	No	No	Yes	No
**Used repeatedly interfered with major role obligations (at work, school, or home)**	No	No	No	No	No	No	No	No	No	No
**Kept using despite knowing it was causing or worsening social or interpersonal problems**	No	No	No	No	No	Yes	No	No	No	No
**Gave up or reduced important social, occupational, or recreational activities because of use**	No	No	No	No	No	No	No	No	No	No
**Repeatedly used in situations where it was physically hazardous**	No	No	No	No	No	No	No	No	No	No
**Kept using despite knowing it was causing or worsening physical or psychological problems**	No	No	No	No	No	Yes	No	No	No	No
**Needed to use larger amounts just to feel the same effect**	No	Yes	No	No	Yes	Yes, but not in past year	No	Yes	Yes, but not in past year	Yes, but not in past year
**Kept using same amount, but didn't feel it as much**	No	Yes, but not in past year	No	No	Yes	Yes	Yes, but not in past year	Yes	Yes	Yes, but not in past year
**Had physical or psychological withdrawal symptoms during times they stopped using**	No	Yes	No	Yes, but not in past year	Yes, but not in past year	Yes	No	No	Yes	No
**Kept using to avoid withdrawal symptoms**	No	Yes, but not in past year	No	Yes, but not in past year	Yes	Yes	No	No	No	No
**KUD Severity (Lifetime)**	None	Moderate	None	Mild	Moderate	Severe	None	Mild	Moderate	None
**KUD Severity (Past Year)**	None	Moderate	None	None	Mild	Severe	None	Mild	Mild	None

Adverse effects upon taking “too much” kratom varied, but the interviews converge upon the point that a person can consume enough kratom to make them uncomfortable at a minimum, though among these and other users, not alarmed enough to seek medical treatment (Garcia-Romeu et al., 2020). However, based on the independently reported descriptions by several of the participants, it is striking that a constellation of symptoms (“the wobbles”) emerged more than once that might indicate that some participants were experiencing signs of serotonergic excess, including agitation, diaphoresis, lower extremity hyperreflexia or clonus (“restless legs”), and possibly even ocular clonus (eyes that were “jittery,” “twitch[y],” “wobbling,” “wiggling,” or “wouldn’t really stay steady”). To our knowledge, there are no published cases of kratom toxicity describing such objective signs of serotonergic excess, which we suspect is most likely because people with kratom toxicity are not being examined by clinicians for hyperreflexia or clonus, assuming such patients present for medical attention at all. However, given these suggestive self-reports along with laboratory findings that at least two kratom alkaloids (paynantheine and speciogynine) are high affinity 5HT-1A receptor ligands, we believe that high doses of kratom may contribute to the development of mild to moderate serotonin syndrome. Clinicians caring for such patients should therefore be alert to this possibility and examine them for signs of serotonergic excess.

Participants did acknowledge the unknown of kratom’s long-term effects, with at least one expressing enough concern to quit, though most emphasized benefits presently outweighing perceived risk. Among those interviewed here, the story of kratom is one in which this natural product helps them get through the day productively, while feeling good but not impaired. This perspective is understandable among those who did not experience any perceived negative outcome or apparent psychosocial impairment. Yet, therein lies the ambiguity. There *are* admitted unknowns.

These unknowns are the other striking feature of participants’ stories. Inherent in their accounts is an uncertainty about what they are taking, where it comes from, how it is processed into products, what the product contains by way of kratom alkaloids, and how these factors affect their health over time. The subjective benefits from kratom were articulated because they are derived from an accessible pharmacological phenomenology. The latent harms, rather than acute adverse experiences, are by their nature inaccessible for self-report. However, it is noteworthy that despite the lack of information about kratom products, the participants regularly using them reasoned that because *Mitragyna speciosa*, from which kratom products are derived, is a plant, and plants are natural, that kratom is therefore less dangerous than pharmaceuticals or illicit drugs when used alone. We know that this is not always the case (Jordan et al., 2010), but among insular groups who use certain plant-based supplements or putative natural products, risks may be minimized as part of the broader marketing strategy. That kratom products are marketed as supplements likely reinforces this conceptualization of kratom as an herbal or alternative medicine. This perceived relative safety is underscored by the widely endorsed belief that one cannot overdose on kratom, particularly powder or tea. Yet, extracts and high doses of any product were acknowledged as warranting caution.

An inescapable limitation of this narrative case series is that our interviewees all lived in one region within the US. Kratom is used around the world, and a full picture of its role in people’s lives requires an international perspective. One important difference between kratom use in southeast Asia and elsewhere is that, in southeast Asia, most kratom preparations use fresh leaves, which have little or none of the alkaloid 7-hydroxymitragynine, present in the dried products sold commercially in the US and elsewhere (Zhang et al., 2022). This and other elements of processing make kratom at least partly a different pharmacological entity outside southeast Asia. Nonetheless, kratom is used in southeast Asia for purposes broadly similar to those reported by our interviewees (energy, analgesia, general psychological wellbeing) (Singh et al., 2015), and its use sometimes leads to problems that are also broadly similar to those reported here (Singh et al., 2019).

Ultimately, the narratives presented here have been shaped by broader narratives about kratom within kratom-using communities, advocacy groups, and the industry (though not uniformly). Moreover, the fact that participants were recruited from websites frequented by people using kratom (e.g., Reddit) may have contributed to their having more nuanced understanding of kratom than someone who purchases it from a local shop or convenience store. This is evidenced by the repeated discussion of optimal dose ranges, strain differences, and convergence of wording regarding adverse effects (e.g., wobbles). Nevertheless, these influences do not delegitimize their lived experiences, so much as contextualize them. We can only understand US kratom use as part of a larger, complex story related to a ballooning industry, unresolved public policy, and woefully inadequate scientific examination.

## 5 Conclusion

Apart from nonhuman animal studies which do not necessarily translate to real-world kratom use (due to dosing amounts and the fact that isolated alkaloids do not reflect commercially available kratom products or whole leaves), we have few data on which to form conclusions about kratom product use. Survey data have provided a crude scaffold for beginning to assemble an understanding of how kratom products are being used in the US and to what effect (Grundmann, 2017; Smith and Lawson, 2017). Because all participants used at least one substance besides kratom (e.g., coffee, antidepressants, cannabis), accounts should be understood within the broader context of polysubstance use. Specific effects from kratom whole-plant matter and individual alkaloids, and interactions with other substances, will need to be investigated in human laboratory studies. Additional qualitative data can help researchers design measures unique to kratom effects when conducting controlled human laboratory experiments. For example, when assessing kratom withdrawal, researchers and clinicians should likely use the Subjective Opioid Withdrawal Scale and Clinical Opioid Withdrawal Scale, but should also assess for stimulant withdrawal symptoms as kratom alkaloids act on more than the opioid system. Likewise, these conversations suggest that kratom toxicity or overdose may not present as opioidergic in nature, but possibly adrenergic or serotoninergic. In addition to pharmacokinetic studies, which need to be dramatically expanded and include measurement of pharmacodynamics, behavioral pharmacology, and drug discrimination studies that help differentiate opioidergic, serotonergic, and other effect profiles are warranted (Tanna et al., 2022). Until sufficient scientific funding and manpower are harnessed to conduct human laboratory studies on kratom alkaloids, including clinical trials for some of the conditions for which people currently use kratom, we should invite people using kratom to share their expertise on kratom-induced effects via these in-depth qualitative discussions. At present, they remain the experts on this subject. This is both an indictment of the current science and a call for further investigation.

## Data Availability

The datasets presented in this article are not readily available because this is qualitative self-report that includes identifiable information in the digital audio and the transcriptions. We may provide such data with identifiers removed at the discretion of the Principal Investigator. Requests to access the datasets should be directed to kirsten.smith@nih.gov.
