# Supplementary material for: Examining the paradoxical effects of kratom: a narrative inquiry
Source: Front Pharmacol. 2023 May 5;14:1174139. doi: 10.3389/fphar.2023.1174139 (PMC10196254; doi:10.3389/fphar.2023.1174139)
Supplement: Supplementary file 1 [file DataSheet1.PDF]

Narrative Inquiry Interview Guide for Kratom-using Adults

1. How did you first learn about kratom?

2. What were the circumstances or factors that influenced your decision to try kratom for the first time?

3. Why have you kept using kratom?

4. What are the ways that has kratom been impactful in your life?

5. What are some of the beneficial experiences or affects that you have received from kratom?
5a. Have these changed over time?

6. What are some of the unhelpful or unwanted experiences or affects that you have received from kratom?
6a. Have these changed over time?

7. Is there a time that you've ever tried to quit using or reduce kratom use and, if so, can you describe what that was like?

8. Looking back at your relationship with kratom, are there any things that you would change or do differently?

9. For someone who has never heard of kratom what would you tell them are the most important things that they should know about kratom?

10. Is there anything else you would like to share?
